# Initial in-hospital heart rate is associated with long-term survival in patients with acute ischemic stroke

**DOI:** 10.1007/s00392-021-01953-5

**Published:** 2021-10-23

**Authors:** Jiann-Der Lee, Ya-Wen Kuo, Chuan-Pin Lee, Yen-Chu Huang, Meng Lee, Tsong-Hai Lee

**Affiliations:** 1 Department of Neurology, Chiayi Chang Gung Memorial Hospital, Chiayi, and College of Medicine, Chang Gung University, Taoyuan, Taiwan; 2 Department of Nursing, Chang Gung University of Science and Technology, Chiayi Campus, Chiayi, Taiwan; 3 Health Information and Epidemiology Laboratory, Chang Gung Memorial Hospital, Chiayi, Taiwan; 4 Department of Neurology, Linkou Chang Gung Memorial Hospital, Taoyuan, and College of Medicine, Chang Gung University, Taoyuan, Taiwan

**Keywords:** Heart rate, Acute ischemic stroke, Mortality, Survival

## Abstract

**Aims:**

Increased heart rate has been associated with stroke risk and outcomes. The purpose of this study was to explore the long-term prognostic value of initial in-hospital heart rate in patients with acute ischemic stroke (AIS).

**Methods:**

We analyzed data from 21,655 patients with AIS enrolled (January 2010–September 2018) in the Chang Gung Research Database. Mean initial in-hospital heart rates were averaged and categorized into 10-beat-per-minute (bpm) increments. The primary and secondary outcomes were all-cause mortality and cardiovascular death. Hazard ratios (HRs) and 95% confidence intervals (CIs) were estimated using multivariable adjusted Cox proportional hazard models, using the heart rate < 60 bpm subgroup as the reference.

**Results:**

The adjusted HRs for all-cause mortality were 1.23 (95% CI 1.08–1.41) for heart rate 60–69 bpm, 1.74 (95% CI 1.53–1.97) for heart rate 70–79 bpm, 2.16 (95% CI 1.89–2.46) for heart rate 80–89 bpm, and 2.83 (95% CI 2.46–3.25) for heart rate ≥ 90 bpm compared with the reference group. Likewise, heart rate ≥ 60 bpm was also associated with an increased risk of cardiovascular death (adjusted HR 1.18 [95% CI 0.95–1.46] for heart rate 60–69 bpm, 1.57 [95% CI 1.28–1.93] for heart rate 70–79 bpm, 1.98 [95% CI 1.60–2.45] for heart rate 80–89 bpm, and 2.36 [95% CI 1.89–2.95] for heart rate ≥ 90 bpm).

**Conclusions:**

High initial in-hospital heart rate is an independent predictor of all-cause mortality and cardiovascular death in patients with AIS.

**Graphical abstract:**

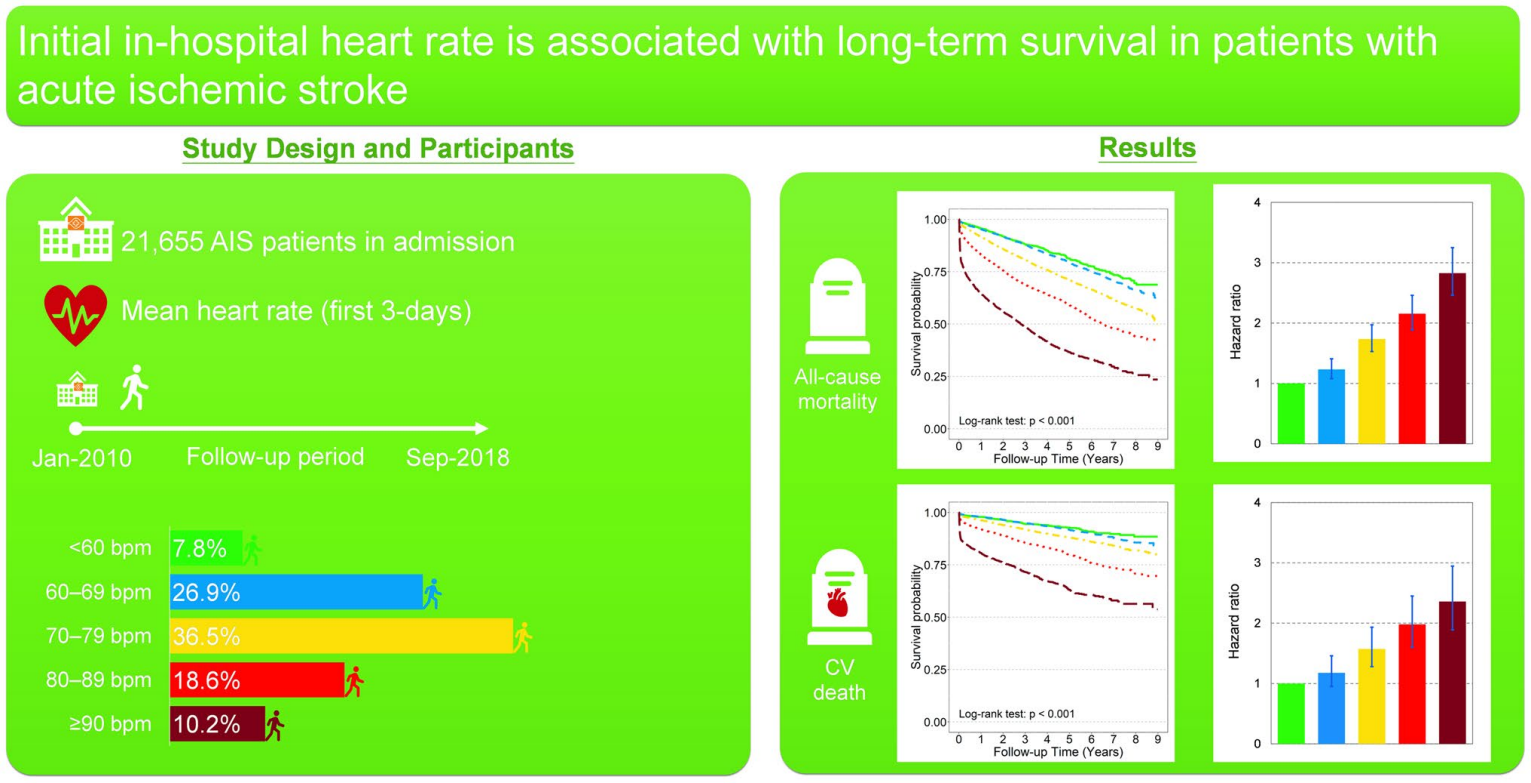

**Supplementary Information:**

The online version contains supplementary material available at 10.1007/s00392-021-01953-5.

## Introduction

Despite improvements in secondary prevention treatment, the incidence rates of mortality and recurrent cardiovascular events post-stroke remain high [1–3]. This indicates that many risk factors have yet to be identified.

A slower heart rate is associated with greater longevity in many mammal species [4]. Heart rate is a vital sign which varies according to the physical needs of the body. Heart rate also reflects the balance between sympathetic and parasympathetic tone to the heart, and it has been shown to be a predictor of cardiovascular and all-cause mortality in the general population and in patients with cardiovascular disease [5–12]. In a review article of 18 epidemiological studies, Aboyans and Criqui reported an increase in mortality rate of 30–50% for every 20-beat-per-minute (bpm) increase in resting heart rate [13]. However, the importance of heart rate is often overlooked. Although experimental studies have suggested that lowering the heart rate may protect against cerebral ischemia by reducing oxidative stress and improving endothelial function [14], whether a lower initial in-hospital heart rate is associated with a better prognosis in patients with acute ischemic stroke (AIS) has yet to be elucidated. Therefore, the objective of this study was to evaluate the relationship between mean initial in-hospital heart rate and long-term mortality in a large population of AIS patients with an extended follow-up.

## Methods

We conducted this retrospective cohort study using data from the Chang Gung Research Database [15], the largest multi-institutional electronic medical records collection in Taiwan. All patients who had an AIS (International Classification of Diseases, 9th Revision, Clinical Modification codes 433.01, 433.11, 433.21, 433.31, 433.81, 433.91, 434.01, 434.11, 434.91; International Classification of Diseases, 10th Revision, Clinical Modification [ICD-10] code I63) in the first two discharge diagnoses [16, 17] between January 2010 and September 2018, and were admitted to one of the seven branch hospitals of Chang Gung Healthcare System were accrued consecutively in this study. Key demographic and clinical characteristics were collected, including stroke severity as assessed using the claims-based stroke severity index (SSI). The SSI was then converted to the National Institutes of Health Stroke Scale score using the equation: estimated National Institutes of Health Stroke Scale (eNIHSS) = 1.1722 × SSI − 0.7533 [18]. Measurements of height, body weight, systolic blood pressure (SBP), diastolic blood pressure (DBP), heart rate, creatinine, alanine aminotransferase (ALT), glycated hemoglobin (HbA1c), and lipid profiles were obtained from the records of the enrolled patients. Heart rate, SBP, and DBP were measured with the patients recumbent after 5 min rest and were recorded using an automated oscillometric device (DINAMAP ProCare 100, GE Medical Systems, Milwaukee, WI, USA) or a bedside patient monitor (IntelliVue MP60, Philips Medical System, Boeblingen, Germany). If the pulse was irregular, heart rates were measured by palpating the radial pulse over a period of 60 s. The mean heart rate was derived from recorded vital sign values in the first 3 days of hospitalization. Estimated glomerular filtration rate (eGFR) was determined using the Modification of Diet in Renal Disease equation as follows: eGFR (mL/min/1.73 m^2^) = 186 × (serum creatinine)^−1.154^ × (age)^−0.203^ × 0.742 (if female) [19]. Chronic kidney disease (CKD) was classified into five stages: stage 1 (eGFR ≥ 90), stage 2 (eGFR 60–89), stage 3 (eGFR 30–59), stage 4 (eGFR 15–29), and stage 5 (eGFR < 15) (all eGFR in mL/min/1.73 m^2^) [20].

**Fig. 1:**
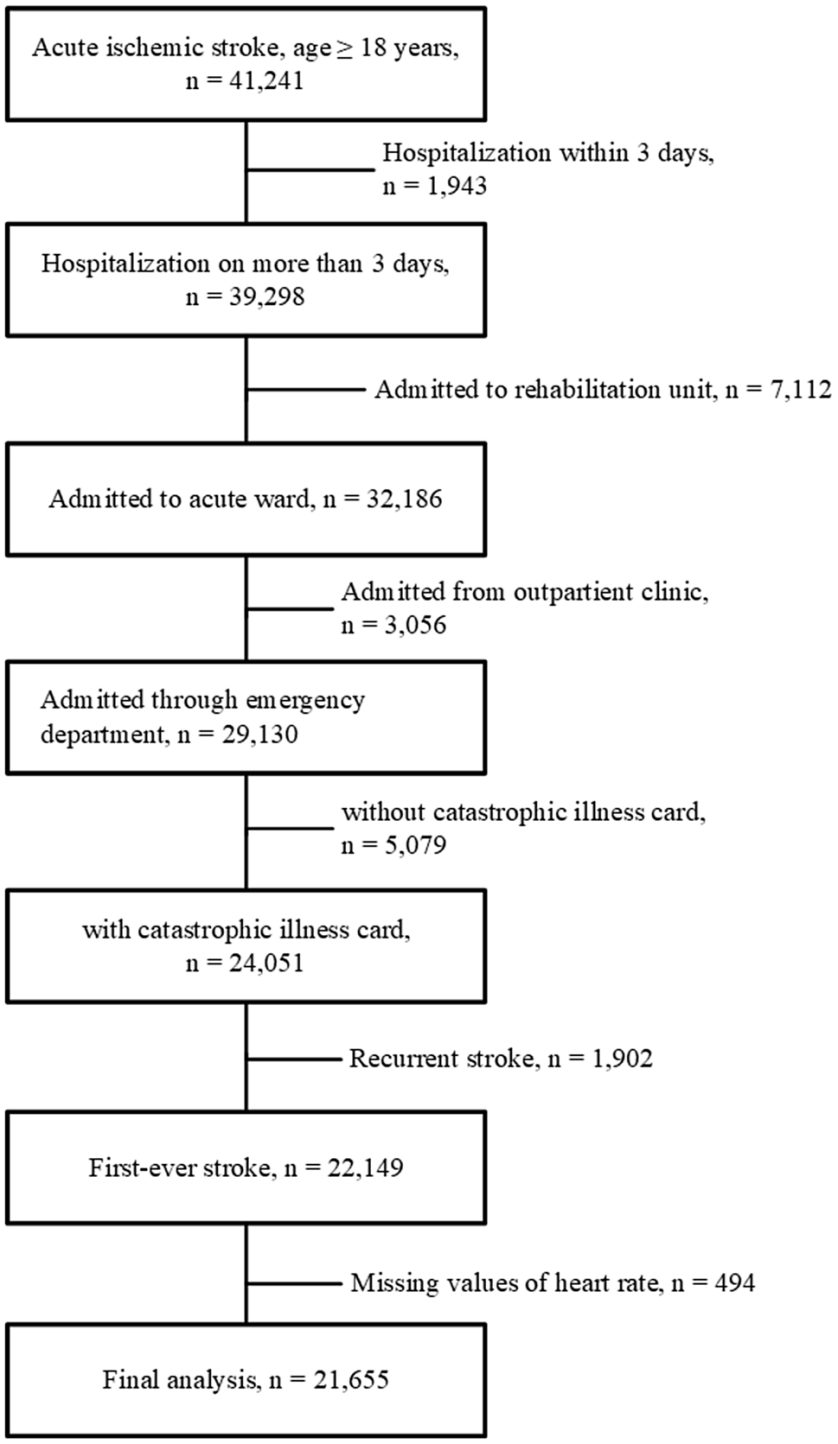
Flowchart of patient selection

The original cohort consisted of 41,241 patients aged ≥ 18 years. The exclusion criteria were patients: (1) hospitalized for less than 3 days; (2) admitted to a rehabilitation unit; (3) admitted through outpatient clinics; (4) with recurrent stroke during the study period; and (5) with less than one record of heart rate per day in the first 3 days of hospitalization. In addition, those without catastrophic illness cards were also excluded (beneficiaries of the National Health Insurance system in Taiwan would have been given a catastrophic illness card for 1 month because of their AIS; the National Health Insurance system in Taiwan covers more than 99.9% of the entire population) [21]. Finally, the data of 21,655 patients were used for the analysis (Fig. 1). The study was conducted in accordance with the Declaration of Helsinki and local ethical approval was obtained.

### Outcomes

The primary outcome was all-cause mortality, and the secondary outcome was cardiovascular death. We linked to the National Registry of Deaths Database provided by the Ministry of Health and Welfare in Taiwan from January 1, 2010 to December 31, 2018. The database includes death certificates coded using ICD-10 codes. Cardiovascular diseases were classified as ICD-10 codes I00–I99.

### Statistical analysis

Descriptive statistics are presented as number (percentage) for categorical data and mean (standard deviation) and median (interquartile range) for continuous data. The patients were classified into five subgroups according to mean heart rate (heart rate < 60, 60–69, 70–79, 80–89, and ≥ 90 bpm). Differences between the groups were tested using the Kruskal–Wallis rank test for continuous data and the Chi-square test for categorical data. In addition to crude hazard ratios (HRs), adjusted HRs and 95% confidence intervals (CIs) were calculated with reference to the lowest risk group and estimated after adjusting for potential confounding factors in the Cox proportional hazard models. Model 1 included age, sex, and eNIHSS, and model 2 included age, sex, eNIHSS, history of hypertension (HTN), diabetes mellitus (DM), dyslipidemia, atrial fibrillation (AF), congestive heart failure (CHF), cancer before admission, smoking status, beta blocking agents, body mass index (BMI), total cholesterol, triglycerides (TGs), CKD stage, ALT, HbA1c, mean SBP, and mean DBP.

Data analysis was conducted without imputing missing data. Variables with missing data were classified into a missing data category to minimize the effect of the missing data in the analysis.

Interactions between mean heart rate and age, sex, eNIHSS, history of HTN, DM, dyslipidemia, AF, CHF, cancer before admission, smoking status, beta blocking agents, BMI, total cholesterol, TGs, CKD stage, ALT, HbA1c, mean SBP, and mean DBP at baseline were tested. Subgroup analyses were performed with heart rate as a continuous variable and when interactions were significant even after adjusting for the same variables as in the Cox proportional hazard model (model 2). HRs and 95% CIs for each subgroup were calculated for every one standard deviation increment in heart rate. A Cox model with restricted cubic spline smoothing technique was used to explore the overall trend of risks through the range of mean heart rate values. All analyses were performed with SAS (version 9.4, Cary, NC, USA) and R (version 4.0).

## Results

### Baseline characteristics

A total of 21,655 adult patients with AIS were included in the analysis (mean age, 67.37 ± 12.92 years; 61.88% males). The mean SBP and DBP were 151.04 ± 19.64 and 84.81 ± 11.17 mmHg, respectively, and the mean heart rate was 74.84 ± 11.27 bpm. There were 436,376 measurements of heart rate in this study. The median number of measurement per patient was 13 (interquartile range 9–18). The demographic data and baseline characteristics of the overall patient cohort and for each 10-bpm-increment heart rate subgroup are given in Table 1. After a median follow-up of 3.2 years (interquartile range 1.4–5.6 years), 6345 patients (29.3%) met the primary outcome (all-cause mortality) and 2584 patients (11.9%) met the secondary outcome (cardiovascular death). Compared to the patients with a higher mean heart rate, those with a lower mean heart rate were more likely to be male, current smokers, without using beta blocking agent, to not have HTN or DM, and to have dyslipidemia, lower eNIHSS score, lower baseline incidence of AF and CHF, lower prevalence of cancer, and lower baseline eGFR level.

**Table 1:** Demographic and baseline characteristics of the overall cohort stratified by mean heart rate

	Number of patients	Mean heart rate						*p* value
		Total (*N* = 21,655)	< 60 bpm (*N* = 1680)	60–69 bpm (*N* = 5834)	70–79 bpm (*N* = 7914)	80–89 bpm (*N* = 4027)	≥ 90 bpm (*N* = 2200)	
Age (year)	21,655							< 0.001
Mean (SD)		67.37 (12.92)	67.84 (11.53)	66.76 (12.27)	66.73 (12.91)	67.88 (13.59)	69.96 (14.02)	
Median (Q1, Q3)		68.00 (59.00, 77.00)	68.00 (60.00, 76.00)	67.00 (59.00, 76.00)	67.00 (58.00, 77.00)	69.00 (59.00, 78.00)	72.00 (61.00, 81.00)	
Male	21,655	13,400 (61.88%)	1224 (72.86%)	3829 (65.63%)	4902 (61.94%)	2262 (56.17%)	1183 (53.77%)	< 0.001
Stroke severity	21,655							< 0.001
Mild (eNIHSS < 6)		14,393 (66.47%)	1334 (79.40%)	4531 (77.67%)	5647 (71.35%)	2265 (56.25%)	616 (28.00%)	
Moderate (eNIHSS 6–13)		3886 (17.95%)	241 (14.35%)	901 (15.44%)	1436 (18.15%)	876 (21.75%)	432 (19.64%)	
Severe (eNIHSS > 13)		3376 (15.59%)	105 (6.25%)	402 (6.89%)	831 (10.50%)	886 (22.00%)	1152 (52.36%)	
Body mass index (kg/m^2^)	14,966							< 0.001
Mean (SD)		24.84 (4.24)	24.79 (3.78)	24.97 (4.01)	25.02 (4.31)	24.73 (4.39)	24.21 (4.59)	
Median (Q1, Q3)		24.49 (22.06, 27.18)	24.55 (22.43, 27.06)	24.65 (22.37, 27.17)	24.68 (22.23, 27.40)	24.34 (21.87, 27.15)	23.81 (21.19, 26.67)	
Hypertension	22,149	12,276 (56.69%)	895 (53.27%)	3191 (54.70%)	4469 (56.47%)	2441 (60.62%)	1280 (58.18%)	< 0.001
Diabetes mellitus	22,149	8680 (40.08%)	468 (27.86%)	2013 (34.50%)	3362 (42.48%)	1879 (46.66%)	958 (43.55%)	< 0.001
Dyslipidemia	22,149	9396 (43.39%)	827 (49.23%)	2791 (47.84%)	3545 (44.79%)	1591 (39.51%)	642 (29.18%)	< 0.001
Atrial fibrillation	22,149	3551 (16.40%)	186 (11.07%)	666 (11.42%)	1071 (13.53%)	894 (22.20%)	734 (33.36%)	< 0.001
Congestive heart failure	22,149	1168 (5.39%)	59 (3.51%)	214 (3.67%)	385 (4.86%)	271 (6.73%)	239 (10.86%)	< 0.001
History of cancer before admission	21,655	1452 (6.71%)	103 (6.13%)	307 (5.26%)	494 (6.24%)	299 (7.42%)	249 (11.32%)	< 0.001
Current smoker	21,655	5967 (27.55%)	618 (36.79%)	1885 (32.31%)	2087 (26.37%)	902 (22.40%)	475 (21.59%)	< 0.001
Beta blocking agent user	21,655	4074 (18.81%)	230 (13.69%)	999 (17.12%)	1448 (18.30%)	845 (20.98%)	552 (25.09%)	< 0.001
Total cholesterol (mmol/L)	19,576							< 0.001
Mean (SD)		4.63 (1.12)	4.56 (1.01)	4.64 (1.03)	4.67 (1.10)	4.63 (1.26)	4.44 (1.26)	
Median (Q1, Q3)		4.53 (3.88, 5.25)	4.50 (3.88, 5.12)	4.55 (3.96, 5.22)	4.58 (3.93, 5.30)	4.53 (3.83, 5.28)	4.32 (3.62, 5.12)	
Triglyceride (mmol/L)	19,572							< 0.001
Mean (SD)		1.51 (1.13)	1.39 (0.89)	1.49 (0.92)	1.55 (1.17)	1.57 (1.30)	1.38 (1.30)	
Median (Q1, Q3)		1.24 (0.90, 1.77)	1.22 (0.88, 1.67)	1.26 (0.91, 1.76)	1.29 (0.93, 1.83)	1.24 (0.89, 1.84)	1.11 (0.80, 1.59)	
CKD	21,655							< 0.001
Stage 1		5430 (25.08%)	366 (21.79%)	1442 (24.72%)	1991 (25.16%)	1050 (26.07%)	581 (26.41%)	
Stage 2		6323 (29.20%)	548 (32.62%)	1810 (31.03%)	2261 (28.57%)	1090 (27.07%)	614 (27.91%)	
Stage 3–5		4838 (22.34%)	290 (17.26%)	1021 (17.50%)	1689 (21.34%)	1095 (27.19%)	743 (33.77%)	
ALT (U/L)	20,873							0.032
Mean (SD)		26.57 (25.73)	25.33 (20.98)	26.22 (21.82)	26.24 (20.91)	27.04 (35.60)	28.76 (32.04)	
Median (Q1, Q3)		21.00 (16.00, 29.00)	21.00 (16.00, 28.00)	21.00 (16.00, 29.00)	21.00 (16.00, 30.00)	20.00 (15.00, 29.00)	21.00 (15.00, 31.00)	
HbA1c (%)	11,365							< 0.001
Mean (SD)		6.89 (1.89)	6.37 (1.28)	6.68 (1.71)	6.93 (1.89)	7.17 (2.10)	7.15 (2.16)	
Median (Q1, Q3)		6.10 (5.70, 7.40)	5.90 (5.60, 6.50)	6.00 (5.70, 7.00)	6.20 (5.70, 7.60)	6.30 (5.70, 8.00)	6.30 (5.70, 7.90)	
Mean SBP (mmHg)	21,622							< 0.001
Mean (SD)		151.04 (19.64)	153.40 (20.27)	151.95 (19.52)	150.73 (19.13)	151.58 (20.01)	146.91 (19.98)	
Median (Q1, Q3)		150.11 (137.20, 164.46)	153.14 (139.40, 167.60)	151.28 (138.14, 165.28)	149.22 (137.09, 163.67)	151.11 (137.87, 165.42)	146.06 (132.96, 160.95)	
Mean DBP (mmHg)	21,609							< 0.001
Mean (SD)		84.81 (11.17)	82.56 (10.97)	84.38 (10.94)	85.21 (10.69)	86.02 (11.67)	84.01 (12.28)	
Median (Q1, Q3)		84.27 (77.22, 92.14)	82.18 (75.19, 89.45)	83.85 (76.86, 91.45)	84.41 (78.00, 92.00)	85.53 (78.00, 94.17)	84.00 (75.86, 92.50)	

*bpm* beats per minute; *SD* standard deviation; *Q* quartile; *eNIHSS* estimated National Institute of Health Stroke Scale; *BMI* body mass index; *HTN* hypertension; *DM* diabetes mellitus; *AF* atrial fibrillation; *CHF* congestive heart failure; *CKD* chronic kidney disease; *ALT* alanine aminotransferase; *HbA1c* glycated hemoglobin; *SBP* systolic blood pressure; *DBP* diastolic blood pressure

### Clinical outcomes according to mean heart rate

**Fig. 2:**
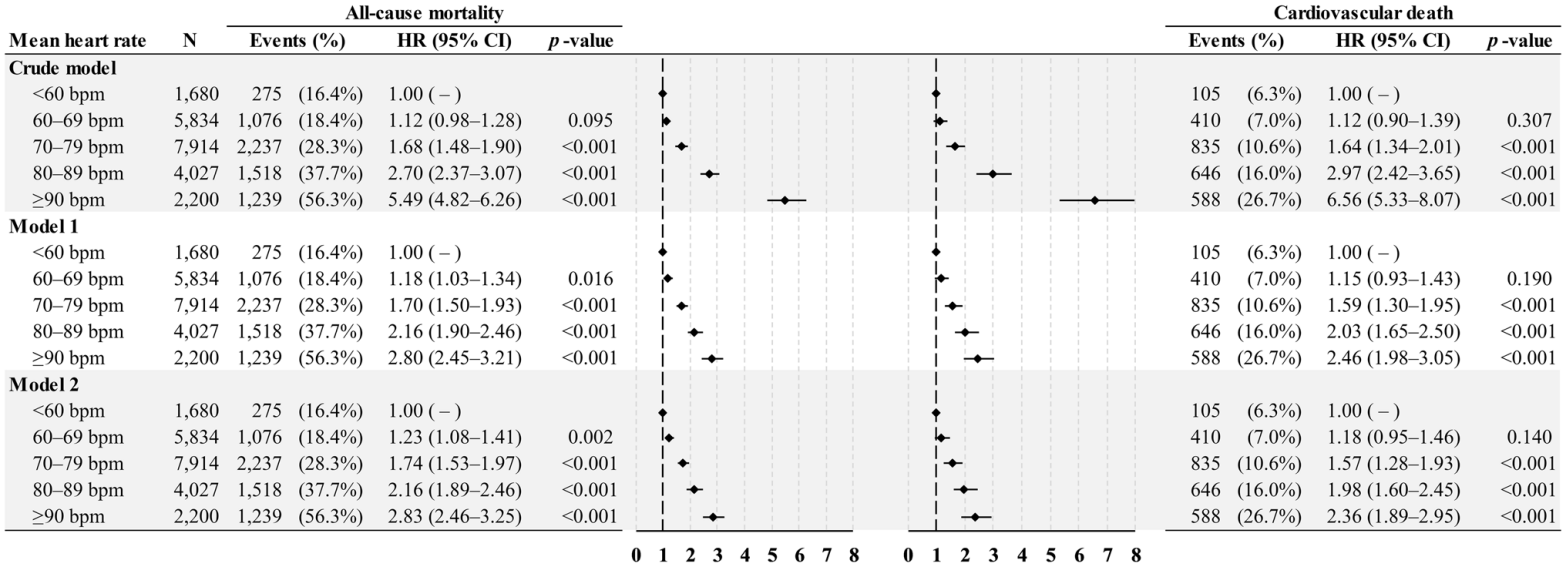
Forest plots of crude and adjusted hazard ratios (95% CIs) of the primary outcome (all-cause mortality) and secondary outcome (cardiovascular death) by mean initial in-hospital heart rate increments. The analyses were adjusted for age and estimated National Institutes of Health Stroke Scale score in model 1, and all of the variables in the fully adjusted model (model 2), including age, sex, estimated National Institutes of Health Stroke Scale score, history of hypertension, diabetes mellitus, dyslipidemia, atrial fibrillation, congestive heart failure, cancer before admission, smoking status, beta blocking agent, body mass index, total cholesterol, triglycerides, chronic kidney disease stage, alanine aminotransferase, glycated hemoglobin, mean systolic blood pressure, and mean diastolic blood pressure. *N* number; *HR* hazard ratio; *CI* confidence interval; *bpm* beats per minute

Crude and adjusted HRs for mean heart rate are given in Fig. 2. Compared with the reference group (mean heart rate < 60 bpm), the adjusted HRs for the all-cause mortality in model 2 was 1.23 (95% CI 1.08–1.41) for mean heart rate 60–69 bpm, 1.74 (95% CI 1.53–1.97) for mean heart rate 70–79 bpm, 2.16 (95% CI 1.89–2.46) for mean heart rate 80–89 bpm, and 2.83 (95% CI 2.46–3.25) for mean heart rate ≥ 90 bpm.

Besides a high mean heart rate, age, male sex, eNIHSS, DM, CHF, history of cancer before admission, and CKD stages 2–5 were all positively associated with the risk of all-cause mortality. Conversely, a history of dyslipidemia, higher BMI, total cholesterol, ALT, and mean DBP levels showed a protective effect (Table 2).

**Table 2:** Multivariable Cox regression models for all-cause mortality and cardiovascular death

Variables	All-cause mortality			Cardiovascular death		
	HR	95% CI	*p* value	HR	95% CI	*p* value
Mean heart rate (ref: < 60 bpm)						
60–69 bpm	1.23	1.08–1.41	0.002	1.18	0.95–1.46	0.140
70–79 bpm	1.74	1.53–1.97	< 0.001	1.57	1.28–1.93	< 0.001
80–89 bpm	2.16	1.89–2.46	< 0.001	1.98	1.60–2.45	< 0.001
≥ 90 bpm	2.83	2.46–3.25	< 0.001	2.36	1.89–2.95	< 0.001
Age	1.041	1.039–1.044	< 0.001	1.038	1.034–1.043	< 0.001
Male (ref: female)	1.38	1.30–1.46	< 0.001	1.35	1.24–1.48	< 0.001
eNIHSS	1.085	1.080–1.090	< 0.001	1.118	1.110–1.125	< 0.001
HTN (ref: without HTN)	0.99	0.94–1.05	0.756	1.08	0.99–1.17	0.074
DM (ref: without DM)	1.17	1.10–1.24	< 0.001	0.92	0.84–1.02	0.105
Dyslipidemia (ref: without dyslipidemia)	0.73	0.69–0.78	< 0.001	0.84	0.76–0.92	< 0.001
AF (ref: without AF)	0.99	0.93–1.06	0.781	1.15	1.05–1.27	0.003
CHF (ref: without CHF)	1.33	1.22–1.45	< 0.001	1.39	1.22–1.58	< 0.001
Cancer (ref: without Cancer)	2.13	1.97–2.30	< 0.001	0.99	0.84–1.16	0.869
Smoker (ref: non-smoker)	0.98	0.92–1.05	0.598	0.98	0.88–1.10	0.769
Beta blocking agents user (ref: non-user)	0.98	0.92–1.05	0.598	1.07	0.98–1.18	0.133
BMI (ref: ≥ 18.5, < 24)						
< 18.5	1.40	1.25–1.57	< 0.001	1.39	1.17–1.65	< 0.001
≥ 24, < 27	0.78	0.73–0.85	< 0.001	0.81	0.72–0.92	0.001
≥ 27, < 30	0.74	0.67–0.82	< 0.001	0.87	0.74–1.01	0.070
≥ 30	0.68	0.60–0.79	< 0.001	0.82	0.67–1.01	0.060
Missing	1.07	1.00–1.13	0.053	1.25	1.13–1.38	< 0.001
Total cholesterol (ref: ≤ Q1)						
> Q1, ≤ median	0.87	0.81–0.93	< 0.001	0.88	0.79–0.98	0.024
> median, ≤ Q3	0.86	0.79–0.92	< 0.001	0.82	0.72–0.92	0.001
> Q3	0.88	0.81–0.95	0.002	0.86	0.75–0.98	0.023
Missing	1.08	0.64–1.82	0.774	1.22	0.58–2.59	0.601
Triglyceride (ref: ≤ Q1)						
> Q1, ≤ median	0.96	0.89–1.03	0.251	0.97	0.87–1.08	0.556
> Median, ≤ Q3	0.96	0.88–1.03	0.250	0.97	0.86–1.09	0.567
> Q3	0.98	0.90–1.07	0.640	0.95	0.82–1.09	0.433
Unknown	0.75	0.45–1.27	0.286	0.66	0.31–1.40	0.275
CKD (ref: stage 1)						
Stage 2	1.13	1.06–1.22	0.001	1.25	1.11–1.39	< 0.001
Stage 3–5	1.79	1.66–1.92	< 0.001	1.79	1.61–2.01	< 0.001
Unknown	0.87	0.80–0.95	0.002	0.78	0.68–0.91	0.001
ALT (ref: ≤ Q1)						
> Q1, ≤ median	0.79	0.74–0.84	< 0.001	0.77	0.69–0.86	< 0.001
> Median, ≤ Q3	0.77	0.71–0.83	< 0.001	0.76	0.68–0.85	< 0.001
> Q3	0.88	0.82–0.94	< 0.001	0.83	0.74–0.93	0.001
Unknown	0.86	0.74–0.99	0.037	0.91	0.72–1.14	0.416
HbA1c (ref: ≤ Q1)						
> Q1, ≤ median	0.95	0.86–1.04	0.262	1.04	0.89–1.21	0.609
> Median, ≤ Q3	1.03	0.94–1.14	0.543	1.20	1.03–1.40	0.017
> Q3	1.11	1.00–1.24	0.054	1.18	0.98–1.41	0.076
Unknown	1.13	1.05–1.22	0.001	1.25	1.11–1.40	< 0.001
Mean SBP (ref: < 130 mmHg)						
≥ 130, < 140 mmHg	0.87	0.80–0.95	0.002	0.85	0.74–0.97	0.018
≥ 140, < 150 mmHg	0.87	0.80–0.95	0.003	0.84	0.73–0.96	0.013
≥ 150, < 160 mmHg	0.93	0.85–1.03	0.151	0.86	0.74–1.00	0.045
≥ 160 mmHg	1.09	0.99–1.20	0.091	1.01	0.87–1.18	0.882
Unknown	1.08	0.23–5.13	0.921	0.89	0.14–5.57	0.899
Mean DBP (ref: < 70 mmHg)						
≥ 70, < 80 mmHg	0.83	0.77–0.91	< 0.001	0.86	0.75–0.97	0.018
≥ 80, < 90 mmHg	0.69	0.62–0.75	< 0.001	0.77	0.67–0.89	< 0.001
≥ 90, < 100 mmHg	0.67	0.60–0.75	< 0.001	0.79	0.66–0.94	0.008
≥ 100 mmHg	0.61	0.52–0.71	< 0.001	0.78	0.62–0.99	0.044
Unknown	0.39	0.09–1.71	0.212	0.59	0.11–3.13	0.531

*HR* hazard ratio; *CI* confidence interval; *ref* reference; *bpm* beats per minute; *eNIHSS* estimated National Institute of Health Stroke Scale; *HTN* hypertension; *DM* diabetes mellitus; *AF* atrial fibrillation; *CHF* congestive heart failure; *BMI* body mass index; *Q* quartile; *CKD* chronic kidney disease; *ALT* alanine aminotransferase; *HbA1c* glycated hemoglobin; *SBP* systolic blood pressure; *DBP* diastolic blood pressure

Compared with the reference group (mean heart rate < 60 bpm), the adjusted HRs for cardiovascular death in model 2 were 1.18 (95% CI 0.95–1.46) for mean heart rate 60–69 bpm, 1.57 (95% CI 1.28–1.93) for mean heart rate 70–79 bpm, 1.98 (95% CI 1.60–2.45) for mean heart rate 80–89 bpm, and 2.36 (95% CI 1.89–2.95) for mean heart rate ≥ 90 bpm (Fig. 2). In addition to a higher mean heart rate, age, male sex, eNIHSS, AF, CHF, and CKD stages 2–5 were still associated with cardiovascular death. A history of dyslipidemia, higher BMI, total cholesterol, ALT, and mean DBP levels showed a protective effect (Table 2).

**Fig. 3:**
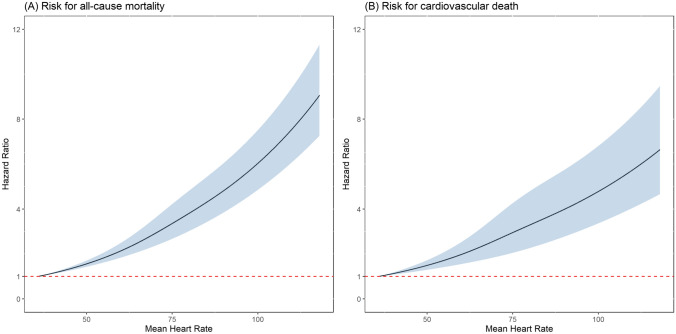
Restricted cubic splines are represented for the associations between mean initial in-hospital heart rate levels and study outcomes (all-cause mortality and cardiovascular death). The analyses were adjusted for all of the variables in the fully adjusted model, including age, sex, estimated National Institutes of Health Stroke Scale score, history of hypertension, diabetes mellitus, dyslipidemia, atrial fibrillation, congestive heart failure, cancer before admission, smoking status, beta blocking agent, body mass index, total cholesterol, triglycerides, chronic kidney disease stage, alanine aminotransferase, glycated hemoglobin, mean systolic blood pressure, and mean diastolic blood pressure

Even after multiple adjustments for potential confounding factors, no J-shaped curve was found for the occurrence of the primary and secondary outcomes. A higher mean heart rate was significantly and continuously associated with increased HRs of all-cause mortality and cardiovascular death (Fig. 3).

### Subgroup analysis

**Fig. 4:**
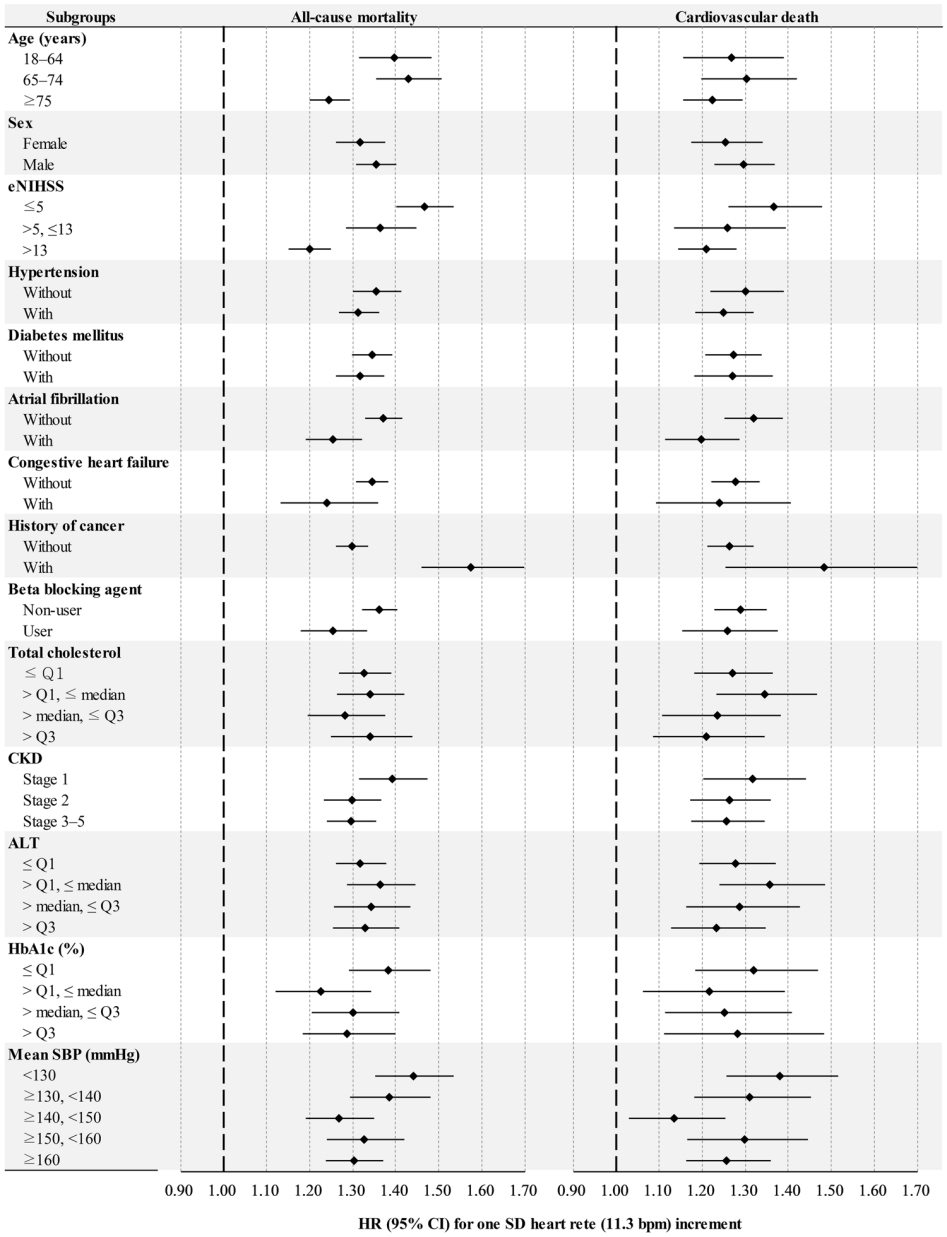
Subgroup analyses of all-cause mortality and cardiovascular death per standard deviation (10.8 beats per minute) of heart rate increment. *eNIHSS* estimated National Institutes of Health Stroke Scale; *CKD* chronic kidney disease; HbA1c, glycated hemoglobin; *SBP* systolic blood pressure; *HR* hazard ratio; *CI* confidence interval; *SD* standard deviation

Interaction analyses are presented in Supplementary Table 1. Significant effect modifications of age, sex, eNIHSS, HTN, DM, AF, CHF, history of cancer before admission, Beta blocking agent, total cholesterol, CKD stage, ALT, HbA1c, and mean SBP at baseline were detected on the relationship between mean heart rate and the primary outcome. An association between mean heart rate and long-term mortality was found in all analyzed subgroups (Fig. 4).

## Discussion

In this study of 21,655 patients with AIS, we found that the mean initial in-hospital heart rate was a predictor of all-cause mortality and cardiovascular death, independently of other known risk factors such as age and stroke severity.

The size of the study cohort allowed us to adjust for two of the strongest predictors of mortality in the multivariable model: age and stroke severity. In the subgroup analysis according to different age and stroke severity groups, the association between initial in-hospital heart rate and long-term mortality was consistent (Fig. 4). The impact of increasing heart rate on all-cause mortality seemed to be more pronounced in the patients younger than 75 years and in those with mild-to-moderate stroke (Fig. 4).

Epidemiological studies have consistently shown that resting heart rate is a predictor of all-cause and cardiovascular mortality in the general population and in patients with cardiovascular disease [22–24]. However, the previous studies have reported inconsistent results about the relationship between heart rate and the clinical outcomes of patients with AIS. The Prevention Regimen for Effectively Avoiding Second Strokes study reported that heart rate was a risk factor for mortality in stroke patients, and, importantly, that a low heart rate was associated with better functional outcomes and lower rates of cognitive impairment after an AIS [25]. In addition, the Gutenberg Health Study reported that both a higher and lower heart rate were associated with a higher risk of mortality [26]. However, Ritter et al. reported that significant tachycardia or bradycardia in AIS did not independently predict the clinical course or outcomes [27]. A higher resting heart rate at admission has also been reported to be independently associated with in-hospital mortality in AIS patients without AF [28]. In addition, Lee et al. reported that in patients with AF hospitalized for AIS, the mean heart rate during the acute period was not associated with stroke recurrence, but was associated with mortality (nonlinear, J-shaped association) [29]. In the present study, mean initial in-hospital heart rate was associated with long-term all-cause and cardiovascular mortality in the overall cohort (Fig. 2), and in the patients with and without AF (Fig. 4). However, the effect of heart rate on long-term mortality seemed to be more pronounced in the patients without AF than in those with AF (Fig. 4). We also found that the long-term survival progressively declined as the level of mean initial in-hospital heart rate increased; however, there was no clear evidence of a J-curve relationship between the mean initial in-hospital heart rate and long-term mortality (Fig. 3).

In the multivariable Cox regression analysis, history of dyslipidemia, higher total cholesterol levels, and higher BMI are associated with improved long-term survival after AIS (Table 2). Although dyslipidemia and high BMI are well-known risk factors for cardiovascular disease, this has not been the case for post-stroke mortality. From several large cohorts, blood lipid levels and BMI have generally been inversely associated with post-stroke mortality [30–32]. There may be unknown protective factors associated with dyslipidemia and high BMI.

Although heart rate is traditionally considered to be a risk factor for cardiovascular disease [33], we found that high initial in-hospital heart rate was a risk factor for both cardiovascular death and all-cause mortality after AIS in the current study. In the long-term follow-up of this study, cardiovascular death and cancer were the leading and secondary causes of death (40.7% and 17.1% of total mortality), respectively. Benetos et al. reported that heart rate was a predictive factor for non-cardiovascular mortality in both men and women in a French population [34]. Another prospective population study also reported that an increased mortality risk associated with a high heart rate was related mainly to diseases of non-cardiovascular or non-malignant origin [35]. However, many studies have demonstrated an association between an elevated heart rate and increased risks of cancer recurrence and mortality [36–38]. For example, a previous meta-analysis found that a higher resting heart rate was associated with increased risks of coronary heart disease, sudden cardiac death, heart failure, AF, stroke, cardiovascular disease, total cancer, and all-cause mortality [39].

Heart rate has been associated with plaque vulnerability, sympathetic hyperactivity, and atherosclerosis [40–42], and therefore, the role of arterial stress related to heart rate in the underlying mechanisms of the progression and clinical manifestations of cardiovascular diseases has gained increasing attention. In this study, the patients with a higher resting heart rate had more cardiovascular risk factors than those in the lowest group (Table 1). Some investigators have also indicated that many cardiovascular risk factors are also related to sympathetic hyperactivity [43–45]. Reduction of heart rate has been shown to delay the progression of coronary atherosclerosis in monkeys [46, 47], and reduce the rate of increase in carotid intima-media thickness in asymptomatic patients [48]. In a model of dyslipidemic mice, chronic heart rate reduction via ivabradine maintained cerebral endothelial function and prevented cerebral artery remodeling [49]. However, whether beta-blockers, calcium-channel blockers, or I(f) channel inhibitor are associated with survival benefit in patients with coronary artery disease is still inconclusive [50–52]. In addition to the above agents, cholinesterase inhibitors can also slow the heart rate via increasing cholinergic effect and show some potential in improving the lifespan [53–55]; however, further studies are needed to investigate whether they can improve survival after AIS. We hope that using appropriate medications to reduce heart rate may play a role in improving the long-term survival after AIS in the future.

### Limitations of this study

The current study has a large sample size and addresses the prognostic implications of initial in-hospital heart rate. However, as with all observational studies, this study has several limitations. First, to collect the vital signs for the first 3 days of hospitalization, patients who were hospitalized for less than 3 days were excluded from the study, and so some patients with mild stroke may have been missed. Second, the link between mean initial in-hospital heart rate and death may not necessarily imply a cause–effect relationship. Third, this study was performed with a population of patients with AIS; therefore, our results may not be applicable to patients with other cardiovascular diseases.

## Conclusion

Heart rate is a simple measurement with important prognostic implications in patients with AIS, and it should no longer be neglected in risk flowcharts.

## Supplementary Information

Below is the link to the electronic supplementary material.

Supplementary file1 (DOCX 14 KB)

## Data Availability

The data supporting the findings of the article are available in the Chang Gung Research Databank at Chang Gung Memorial Hospital, Chiayi Branch. These data can be available after obtaining approval from our local IRB.

## Abbreviations

bpm: Beats per minute
AIS: Acute ischemic stroke
ICD-10: International Classification of Diseases, 10th Revision, Clinical Modification
SSI: Stroke severity index
eNIHSS: Estimated National Institutes of Health Stroke Scale
SBP: Systolic blood pressure
DBP: Diastolic blood pressure
ALT: Alanine aminotransferase
HbA1c: Glycated hemoglobin
eGFR: Estimated glomerular filtration rate
CKD: Chronic kidney disease
CI: Confidence interval
HTN: Hypertension
DM: Diabetes mellitus
AF: Atrial fibrillation
CHF: Congestive heart failure
TG: Triglyceride
BMI: Body mass index

## References

[1] Bronnum-Hansen H, Davidsen M, Thorvaldsen P, Danish MSG (2001). Long-term survival and causes of death after stroke. Stroke.

[2] Singh RJ, Chen S, Ganesh A, Hill MD (2018). Long-term neurological, vascular, and mortality outcomes after stroke. Int J Stroke.

[3] Chen Y, Wright N, Guo Y, Turnbull I, Kartsonaki C, Yang L, Bian Z, Pei P, Pan D, Zhang Y (2020). Mortality and recurrent vascular events after first incident stroke: a 9-year community-based study of 0.5 million Chinese adults. Lancet Glob Health.

[4] Levine HJ (1997). Rest heart rate and life expectancy. J Am Coll Cardiol.

[5] Kannel WB, Kannel C, Paffenbarger RS, Cupples LA (1987). Heart rate and cardiovascular mortality: the Framingham Study. Am Heart J.

[6] Gillman MW, Kannel WB, Belanger A, D'Agostino RB (1993). Influence of heart rate on mortality among persons with hypertension: the Framingham Study. Am Heart J.

[7] Bohm M, Swedberg K, Komajda M, Borer JS, Ford I, Dubost-Brama A, Lerebours G, Tavazzi L, Investigators S (2010). Heart rate as a risk factor in chronic heart failure (SHIFT): the association between heart rate and outcomes in a randomised placebo-controlled trial. Lancet.

[8] Zhao MX, Zhao Q, Zheng M, Liu T, Li Y, Wang M, Yao S, Wang C, Chen YM, Xue H (2020). Effect of resting heart rate on the risk of all-cause death in Chinese patients with hypertension: analysis of the Kailuan follow-up study. BMJ Open.

[9] Saxena A, Minton D, Lee DC, Sui X, Fayad R, Lavie CJ, Blair SN (2013). Protective role of resting heart rate on all-cause and cardiovascular disease mortality. Mayo Clin Proc.

[10] Li K, Yao C, Yang X, Dong L (2017). Effect of resting heart rate on all-cause mortality and cardiovascular events according to age. J Am Geriatr Soc.

[11] Diaz A, Bourassa MG, Guertin MC, Tardif JC (2005). Long-term prognostic value of resting heart rate in patients with suspected or proven coronary artery disease. Eur Heart J.

[12] Kristal-Boneh E, Silber H, Harari G, Froom P (2000). The association of resting heart rate with cardiovascular, cancer and all-cause mortality. Eight year follow-up of 3527 male Israeli employees (the CORDIS Study). Eur Heart J.

[13] Aboyans V, Criqui MH (2006). Can we improve cardiovascular risk prediction beyond risk equations in the physician's office?. J Clin Epidemiol.

[14] Custodis F, Gertz K, Balkaya M, Prinz V, Mathar I, Stamm C, Kronenberg G, Kazakov A, Freichel M, Bohm M (2011). Heart rate contributes to the vascular effects of chronic mental stress: effects on endothelial function and ischemic brain injury in mice. Stroke.

[15] Tsai MS, Lin MH, Lee CP, Yang YH, Chen WC, Chang GH, Tsai YT, Chen PC, Tsai YH (2017). Chang Gung Research Database: a multi-institutional database consisting of original medical records. Biomed J.

[16] Sacco RL, Kasner SE, Broderick JP, Caplan LR, Connors JJ, Culebras A, Elkind MS, George MG, Hamdan AD, Higashida RT (2013). An updated definition of stroke for the 21st century: a statement for healthcare professionals from the American Heart Association/American Stroke Association. Stroke.

[17] Hsieh MT, Hsieh CY, Tsai TT, Wang YC, Sung SF (2020). Performance of ICD-10-CM diagnosis codes for identifying acute ischemic stroke in a national health insurance claims database. Clin Epidemiol.

[18] Sung SF, Hsieh CY, Lin HJ, Chen YW, Chen CH, Kao Yang YH, Hu YH (2016). Validity of a stroke severity index for administrative claims data research: a retrospective cohort study. BMC Health Serv Res.

[19] Levey AS, Bosch JP, Lewis JB, Greene T, Rogers N, Roth D (1999). A more accurate method to estimate glomerular filtration rate from serum creatinine: a new prediction equation. Modification of diet in renal disease study group. Ann Intern Med.

[20] Levey AS, Eckardt KU, Tsukamoto Y, Levin A, Coresh J, Rossert J, De Zeeuw D, Hostetter TH, Lameire N, Eknoyan G (2005). Definition and classification of chronic kidney disease: a position statement from kidney disease: improving global outcomes (KDIGO). Kidney Int.

[21] Cheng TM (2015). Reflections on the 20th anniversary of Taiwan's single-payer national health insurance system. Health Aff (Millwood).

[22] Fox K, Borer JS, Camm AJ, Danchin N, Ferrari R, Lopez Sendon JL, Steg PG, Tardif JC, Tavazzi L, Tendera M (2007). Resting heart rate in cardiovascular disease. J Am Coll Cardiol.

[23] Palatini P (2007). Heart rate as an independent risk factor for cardiovascular disease: current evidence and basic mechanisms. Drugs.

[24] Seccareccia F, Pannozzo F, Dima F, Minoprio A, Menditto A, Lo Noce C, Giampaoli S, Superiore MCAI, di Sanita P (2001). Heart rate as a predictor of mortality: the MATISS project. Am J Public Health.

[25] Bohm M, Cotton D, Foster L, Custodis F, Laufs U, Sacco R, Bath PM, Yusuf S, Diener HC (2012). Impact of resting heart rate on mortality, disability and cognitive decline in patients after ischaemic stroke. Eur Heart J.

[26] Munzel T, Hahad O, Gori T, Hollmann S, Arnold N, Prochaska JH, Schulz A, Beutel M, Pfeiffer N, Schmidtmann I (2019). Heart rate, mortality, and the relation with clinical and subclinical cardiovascular diseases: results from the Gutenberg Health Study. Clin Res Cardiol.

[27] Ritter MA, Rohde A, Heuschmann PU, Dziewas R, Stypmann J, Nabavi DG, Ringelstein BE (2011) Heart rate monitoring on the stroke unit. What does heart beat tell about prognosis? An observational study. BMC Neurol 11:47. 10.1186/1471-2377-11-47 PMC3096901 21524295

[28] Han Q, Zhang C, You S, Zheng D, Zhong C, Dong H, Wang X, Pei S, Cao Y, Liu CF (2020). Resting heart rate and in-hospital mortality in acute ischemic stroke patients with and without atrial fibrillation. Circ J.

[29] Lee KJ, Kim BJ, Han MK, Kim JT, Choi KH, Shin DI, Yeo MJ, Cha JK, Kim DH, Nah HW (2020). Effect of heart rate on stroke recurrence and mortality in acute ischemic stroke with atrial fibrillation. Stroke.

[30] Yeramaneni S, Kleindorfer DO, Sucharew H, Alwell K, Moomaw CJ, Flaherty ML, Woo D, Adeoye O, Ferioli S, de Los Rios La Rosa F (2017). Hyperlipidemia is associated with lower risk of poststroke mortality independent of statin use: a population-based study. Int J Stroke.

[31] Olsen TS, Dehlendorff C, Petersen HG, Andersen KK (2008). Body mass index and poststroke mortality. Neuroepidemiology.

[32] Aparicio HJ, Himali JJ, Beiser AS, Davis-Plourde KL, Vasan RS, Kase CS, Wolf PA, Seshadri S (2017) Overweight, obesity, and survival after stroke in the Framingham Heart Study. J Am Heart Assoc 6:e004721 10.1161/JAHA.116.004721 PMC5669145 28647687

[33] Perret-Guillaume C, Joly L, Benetos A (2009). Heart rate as a risk factor for cardiovascular disease. Prog Cardiovasc Dis.

[34] Benetos A, Rudnichi A, Thomas F, Safar M, Guize L (1999). Influence of heart rate on mortality in a French population: role of age, gender, and blood pressure. Hypertension.

[35] Reunanen A, Karjalainen J, Ristola P, Heliovaara M, Knekt P, Aromaa A (2000). Heart rate and mortality. J Intern Med.

[36] Lee DH, Park S, Lim SM, Lee MK, Giovannucci EL, Kim JH, Kim SI, Jeon JY (2016). Resting heart rate as a prognostic factor for mortality in patients with breast cancer. Breast Cancer Res Treat.

[37] Park J, Kim JH, Park Y, Park SJ, Cheon JH, Kim WH, Park JS, Jeon JY, Kim TI (2018). Resting heart rate is an independent predictor of advanced colorectal adenoma recurrence. PLoS ONE.

[38] Anker MS, Ebner N, Hildebrandt B, Springer J, Sinn M, Riess H, Anker SD, Landmesser U, Haverkamp W, von Haehling S (2016). Resting heart rate is an independent predictor of death in patients with colorectal, pancreatic, and non-small cell lung cancer: results of a prospective cardiovascular long-term study. Eur J Heart Fail.

[39] Aune D, Sen A, O’Hartaigh B, Janszky I, Romundstad PR, Tonstad S, Vatten LJ (2017). Resting heart rate and the risk of cardiovascular disease, total cancer, and all-cause mortality—a systematic review and dose-response meta-analysis of prospective studies. Nutr Metab Cardiovasc Dis.

[40] Dominguez-Rodriguez A, Blanco-Palacios G, Abreu-Gonzalez P (2011). Increased heart rate and atherosclerosis: potential implications of ivabradine therapy. World J Cardiol.

[41] Custodis F, Schirmer SH, Baumhakel M, Heusch G, Bohm M, Laufs U (2010). Vascular pathophysiology in response to increased heart rate. J Am Coll Cardiol.

[42] Palatini P, Julius S (1997). Association of tachycardia with morbidity and mortality: pathophysiological considerations. J Hum Hypertens.

[43] Festa A, D'Agostino R, Hales CN, Mykkanen L, Haffner SM (2000). Heart rate in relation to insulin sensitivity and insulin secretion in nondiabetic subjects. Diabetes Care.

[44] Grynberg A, Ziegler D, Rupp H (1996). Sympathoadrenergic overactivity and lipid metabolism. Cardiovasc Drugs Ther.

[45] Wang A, Liu X, Guo X, Dong Y, Wu Y, Huang Z, Xing A, Luo Y, Jonas JB, Wu S (2014) Resting heart rate and risk of hypertension: results of the Kailuan cohort study. J Hypertens 32:1600–1605; discussion 1605 10.1097/HJH.0000000000000230 24879491

[46] Beere PA, Glagov S, Zarins CK (1984). Retarding effect of lowered heart rate on coronary atherosclerosis. Science.

[47] Kaplan JR, Manuck SB, Adams MR, Weingand KW, Clarkson TB (1987). Inhibition of coronary atherosclerosis by propranolol in behaviorally predisposed monkeys fed an atherogenic diet. Circulation.

[48] Hedblad B, Wikstrand J, Janzon L, Wedel H, Berglund G (2001). Low-dose metoprolol CR/XL and fluvastatin slow progression of carotid intima-media thickness: main results from the beta-blocker cholesterol-lowering asymptomatic plaque study (BCAPS). Circulation.

[49] Bolduc V, Drouin A, Gillis MA, Duquette N, Thorin-Trescases N, Frayne-Robillard I, Des Rosiers C, Tardif JC, Thorin E (2011). Heart rate-associated mechanical stress impairs carotid but not cerebral artery compliance in dyslipidemic atherosclerotic mice. Am J Physiol Heart Circ Physiol.

[50] Cruz Rodriguez JB, Alkhateeb H (2020). Beta-blockers, calcium channel blockers, and mortality in stable coronary artery disease. Curr Cardiol Rep.

[51] Kjekshus JK (1986). Importance of heart rate in determining beta-blocker efficacy in acute and long-term acute myocardial infarction intervention trials. Am J Cardiol.

[52] Fox K, Ford I, Steg PG, Tendera M, Robertson M, Ferrari R, Investigators B (2009). Relationship between ivabradine treatment and cardiovascular outcomes in patients with stable coronary artery disease and left ventricular systolic dysfunction with limiting angina: a subgroup analysis of the randomized, controlled BEAUTIFUL trial. Eur Heart J.

[53] Meguro K, Kasai M, Akanuma K, Meguro M, Ishii H, Yamaguchi S (2014). Donepezil and life expectancy in Alzheimer's disease: a retrospective analysis in the Tajiri Project. BMC Neurol.

[54] Secnik J, Schwertner E, Alvarsson M, Hammar N, Fastbom J, Winblad B, Garcia-Ptacek S, Religa D, Eriksdotter M (2020) Cholinesterase inhibitors in patients with diabetes mellitus and dementia: an open-cohort study of ~23 000 patients from the Swedish Dementia Registry. BMJ Open Diabetes Res Care 8 10.1136/bmjdrc-2019-000833 PMC7039592 31958305

[55] Isik AT, Soysal P, Stubbs B, Solmi M, Basso C, Maggi S, Schofield P, Veronese N, Mueller C (2018). Cardiovascular outcomes of cholinesterase inhibitors in individuals with dementia: a meta-analysis and systematic review. J Am Geriatr Soc.

