# Supplementary material for: Initial in-hospital heart rate is associated with long-term survival in patients with acute ischemic stroke
Source: Clin Res Cardiol. 2021 Oct 23;111(6):651–62. doi: 10.1007/s00392-021-01953-5 (PMC9151537; doi:10.1007/s00392-021-01953-5)
Supplement: Supplementary file 1 — Supplementary file1 (DOCX 14 KB) [file 392_2021_1953_MOESM1_ESM.docx]

**Supplementary Table 1. Interaction analysis for mean heart rate and each variable (for all-cause mortality)**

| Variable | *p*-value |
| --- | --- |
| Age | < 0.001 |
| Sex | 0.029 |
| eNIHSS | < 0.001 |
| HTN | < 0.001 |
| DM | < 0.001 |
| Dyslipidemia | 0.604 |
| AF | < 0.001 |
| CHF | < 0.001 |
| History of cancer | 0.035 |
| Smoking | 0.165 |
| Beta blocking agent user | < 0.001 |
| BMI | 0.184 |
| Total cholesterol | 0.043 |
| Triglyceride | 0.067 |
| CKD stage | < 0.001 |
| ALT | 0.005 |
| HbA1c | 0.001 |
| Mean SBP | < 0.001 |
| Mean DBP | 0.355 |

The *p*-values were estimated by Cox regression model including main effects and 2-factor interaction of mean heart rate and each variable. Abbreviations: eNIHSS, estimated National Institute of Health Stroke Scale; HTN, hypertension; DM, diabetes mellitus; AF, atrial fibrillation; CHF, congestive heart failure; BMI, body mass index; CKD, chronic kidney disease; ALT, alanine aminotransferase; HbA1c, glycated hemoglobin; SBP, systolic blood pressure; DBP, diastolic blood pressure.
